# Triplanar Anteromedial Closing Wedge Tibial Osteotomy in Excessive Slope and Tibial Valgum Patients

**DOI:** 10.1016/j.eats.2024.103400

**Published:** 2024-12-27

**Authors:** Matthieu Ollivier, Shintaro Onishi, Maher Ghandour, Hiroshi Nakayama, Kristian Kley, Julian Fürmetz

**Affiliations:** a Institute for Locomotion, Sainte-Marguerite Hospital, Aix-Marseille University, Marseille, France; b Department of Orthopaedic Surgery, Hyogo College of Medicine, Nishinomiya, Japan; c Department of Orthopedics and Trauma Surgery, Helios University Hospital, University, Wuppertal, Germany; d Orthopaedic Care Center, Harley Street Specialist Hospital, London, U.K.; e BG Trauma Center Murnau, Murnau, Germany

## Abstract

Excessive posterior tibial slope (PTS) is a known risk factor for anterior cruciate ligament (ACL) injury and subsequent failure of ACL reconstruction. While anterior closing wedge osteotomy effectively reduces PTS, it offers limited correction for coronal plane deformities such as tibial valgum. Tibial valgum can occur as a growth abnormality, particularly in skeletally immature patients, even after ACL reconstruction. Addressing both excessive PTS and tibial valgum simultaneously poses a significant challenge due to the lack of an established correction procedure. The triplanar anteromedial closing wedge osteotomy is a demanding surgical technique that addresses this challenge by correcting both sagittal and coronal plane deformities in patients with excessive PTS and tibial valgum. By utilizing patient-specific instrumentation, this complex osteotomy is made simpler and more accurate, allowing for optimal deformity correction. This article describes an open surgical technique for performing anteromedial closing wedge osteotomy using patient-specific instrumentation to reduce recurrent ACL injury risk while maintaining physiologic joint alignment.

Anterior cruciate ligament (ACL) injuries are common in the young athletic population.^1^ Excessive posterior tibial slope (PTS) is known to be an anatomic risk factor for both ACL injuries and subsequent ACL reconstruction (ACLR) failure.^2^ Anterior closing wedge osteotomy (ACWO) of the proximal tibia is an effective technique to reduce the excessive PTS and decrease the ACL graft forces and the risk of ACLR failure.^3^^,^^4^ However, this procedure has a disadvantage of limited capacity of correction in coronal alignment.^5^ A previous study has shown that patients with multiple ACLR failures are more likely to have chondral injuries in the medial compartment.^6^ For varus knee osteoarthritis due to ACL insufficiency, high tibial osteotomy is known as a viable option to correct from varus to slightly valgus alignment.^7^

On the other hand, tibial valgum can occur as a growth abnormality even after ACLR in skeletally immature patients.^8^ Triplanar anteromedial closing wedge osteotomy (AMCWO) is an osteotomy to correct both abnormal PTS and tibial valgum. Herein, we describe a technique for AMCWO using patient-specific instrumentation (PSI) that can accurately correct the observed deformity.

## Surgical Technique

A detailed video of the triplanar AMCWO technique is shown in Video 1. Surgical pearls and pitfalls of this procedure are listed in Table 1.

**Table 1:** Pearls and Pitfalls

Pearls	Pitfalls
- Preoperative planning using both radiographs and CT scans is essential to achieve the intended correction without introducing either loss of correction or iatrogenic secondary deformity.	- PSI is made according to the preoperative CT scan taken in the nonweightbearing supine position. Correction errors, particularly in coronal alignment, may occur without taking into consideration the weightbearing long-leg radiographs.
- Adequate exposure is required to place the PSI guide correctly.	- Failure to place the PSI as planned preoperatively may result in the correction error.
- Verifying the optimal position of the PSI guide under fluoroscopy with reference to the preoperative plan is mandatory.	- Although the PSI guide is used, surgeons should be aware that an optimal osteotomy is necessary to completely close the osteotomy gap.
- Hinge protection wire is used to reduce the risk of hinge fracture.	
- A cortical screw is inserted at the distal aspects of the locking plate to put the compression force into the osteotomy site.	
- It is essential to avoid interfering locking screws with tibial bone tunnel in ACL reconstruction.	

ACL, anterior cruciate ligament; CT, computed tomography; PSI, patient-specific instrumentation.

### Preoperative Planning

**Fig 1:**
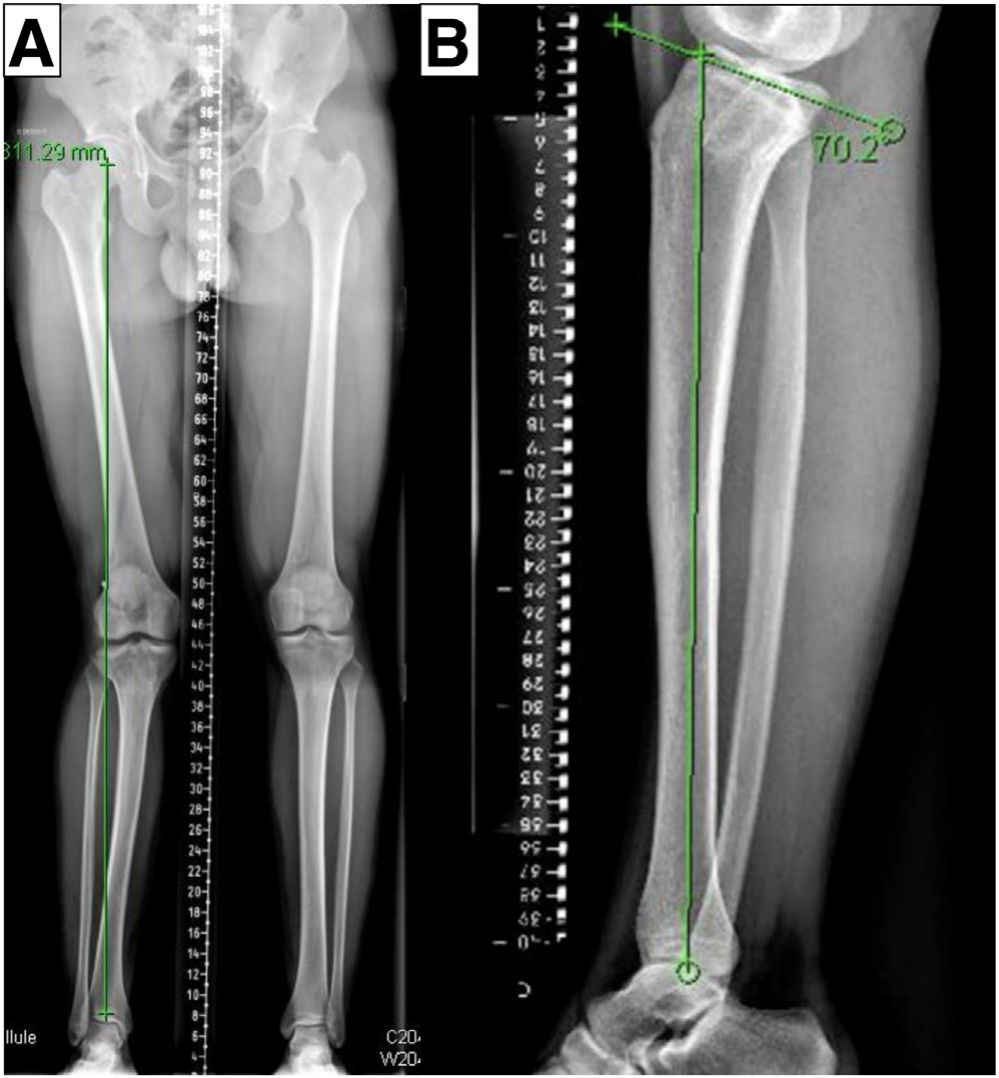
Preoperative long-leg radiographs (right knee). (A) Marked valgus deformity is observed in the right knee. (B) Posterior tibial slope is approximately 20°.

**Fig 2:**
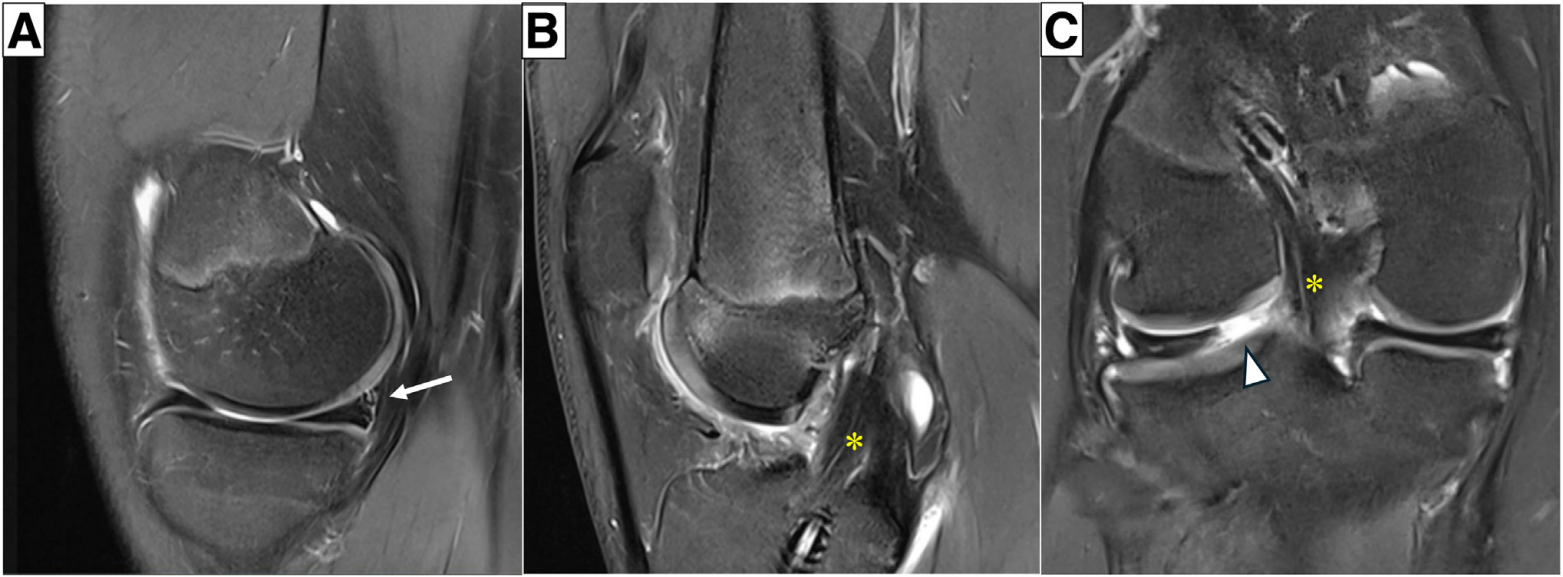
(A) Preoperative magnetic resonance image shows ramp lesion of medial meniscus (white arrow). (B) Previous reconstructed graft is still visible (∗), but tibial bone tunnel was misplaced posteriorly. (C) Coronal view shows posterior root radial tear of the lateral meniscus (arrowhead).

**Fig 3:**
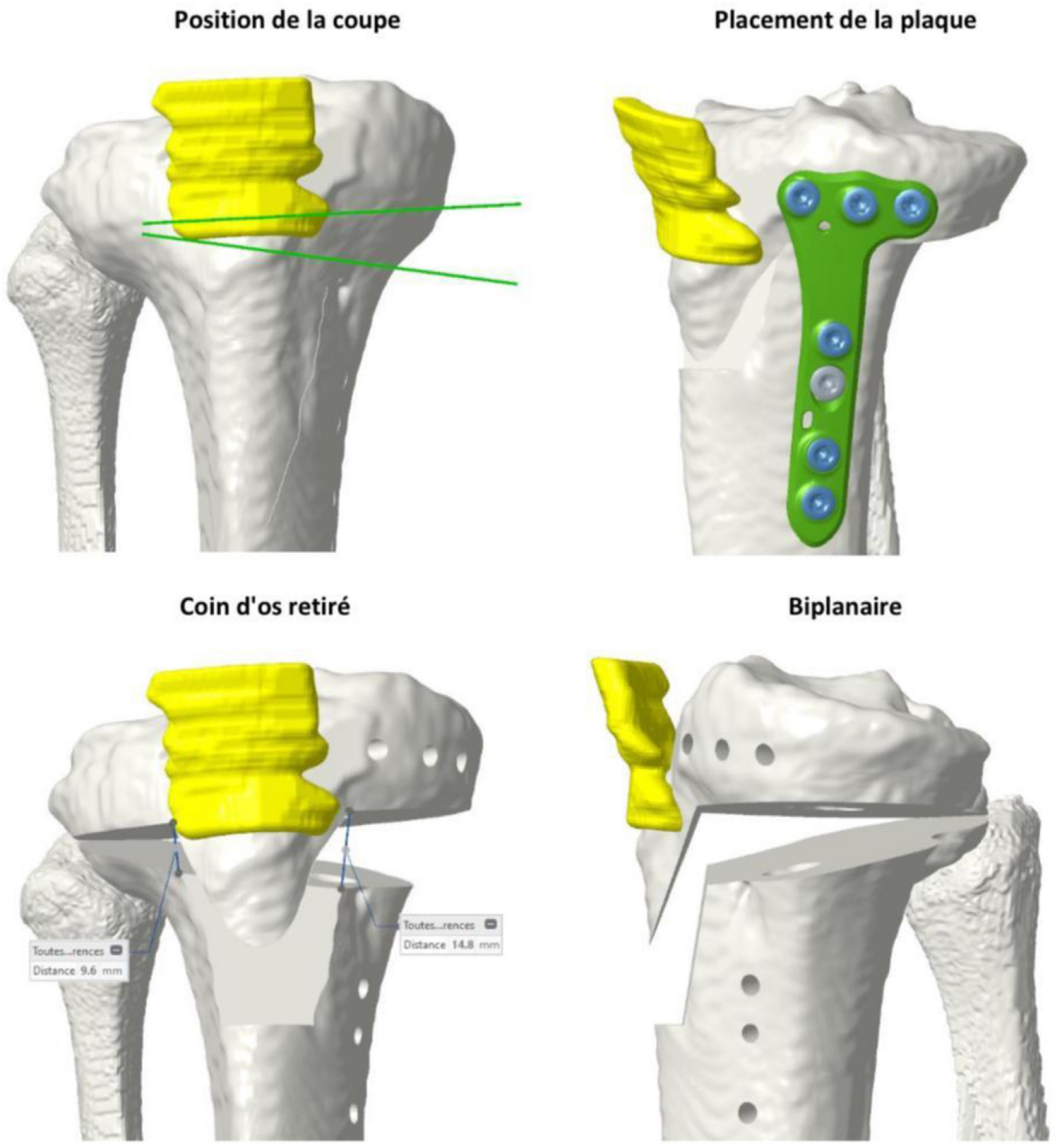
Preoperative planning of osteotomy using patient-specific instrumentation.

Preoperatively, standard radiographs, including weightbearing long-leg radiographs, anteroposterior and lateral views, and computed tomography (CT), are performed. Long-leg radiographs or full-length tibia lateral radiographs are used to assess the coronal and sagittal alignment (Fig 1). In addition, a preoperative magnetic resonance image is required to assess the intra-articular pathology (Fig 2). The PSI guide (Newclip Technics) is made to aim the postoperative medial proximal tibial angle of 87° according to preoperative CT data under the surgeon’s guidance (Fig 3). The CT scan protocol consists of acquiring selective images centered on the femoral head, the knee, and finally the ankle joint. The slice thickness is 0.625 mm for the knee and 2 mm for the hip and ankle, as previously described.^9^ A distal tuberosity osteotomy is planned to prevent unintended changes in patellar height during this procedure. Noteworthy, care should be taken to discriminate the effect of weightbearing on the whole limb alignment between a CT scan taken with a nonweightbearing supine position and a weightbearing long-leg radiograph.

### Anesthesia and Positioning

After either general or spinal anesthesia is administered, an examination under anesthesia is performed. The patient is positioned in a supine position with a nonsterile tourniquet. The knee is placed at 90° of flexion, and diagnostic arthroscopy is then performed.

### Arthroscopic Procedure and Graft Harvest

Arthroscopic intra-articular procedures such as meniscal repair or chondral procedures are performed as needed. If concomitant ACLR is performed, the graft is harvested if using an autograft, and then femoral bone tunnels of both ACLR and lateral tenodesis are created until the osteotomy procedure begins.

### Osteotomy

**Fig 4:**
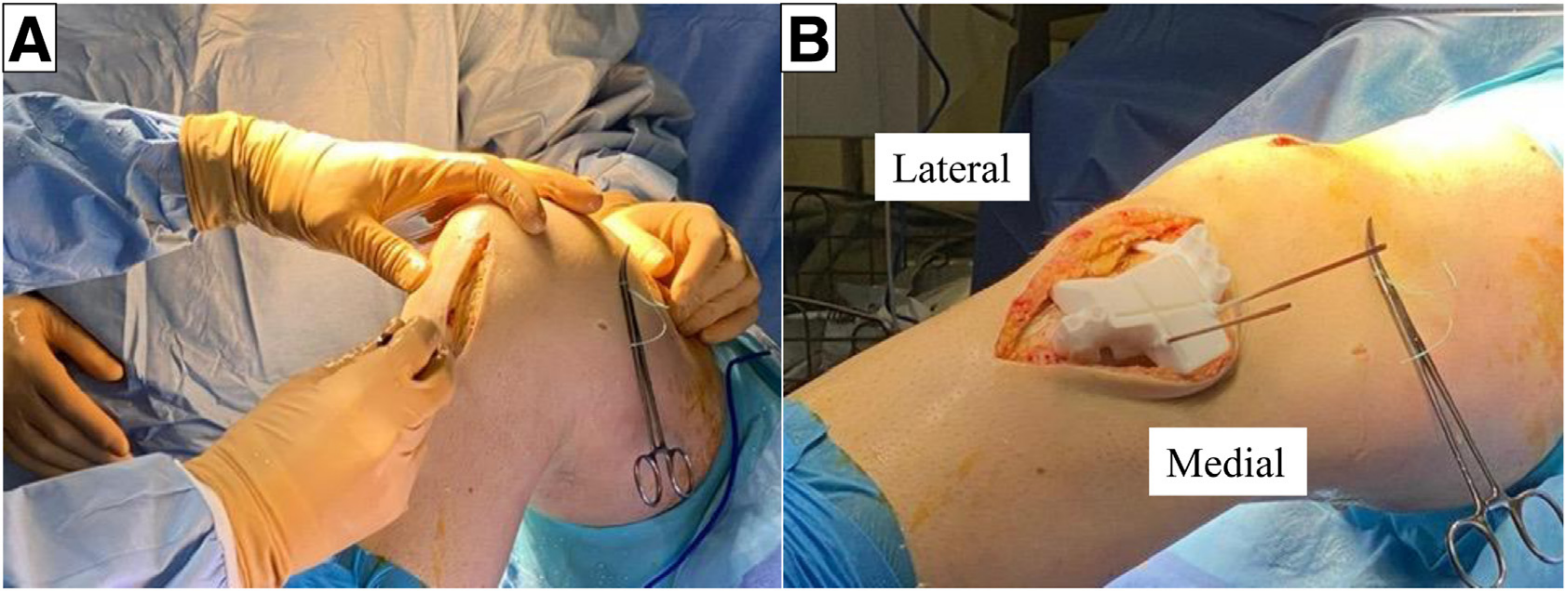
Intraoperative photographs showing exposure to place the patient-specific guide. The patient is positioned in a supine position. The knee is placed at 90° of flexion.

**Fig 5:**
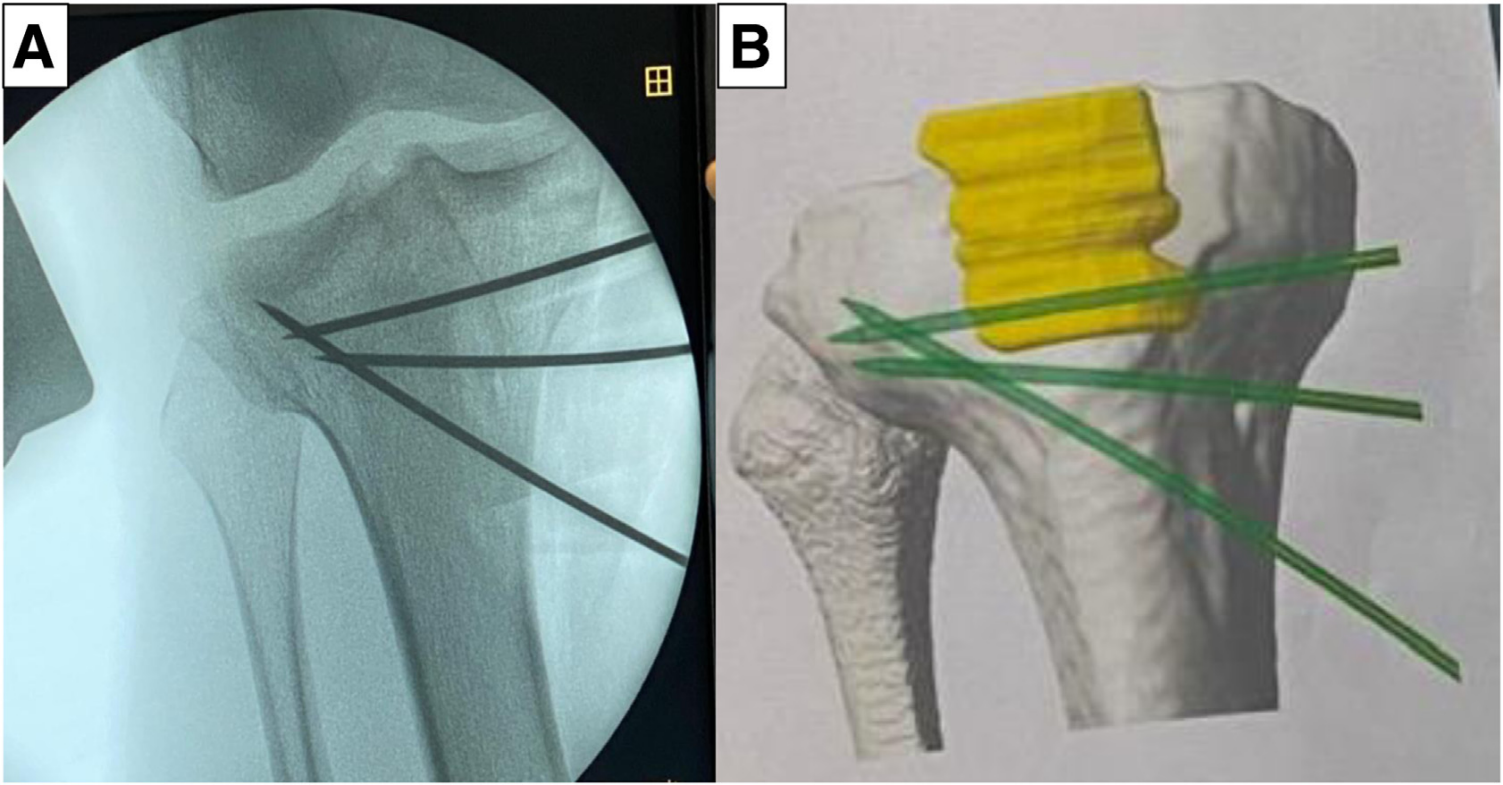
Intraoperative image under fluoroscopy shows optimal location of the guidewires according to the preoperative planning.

**Fig 6:**
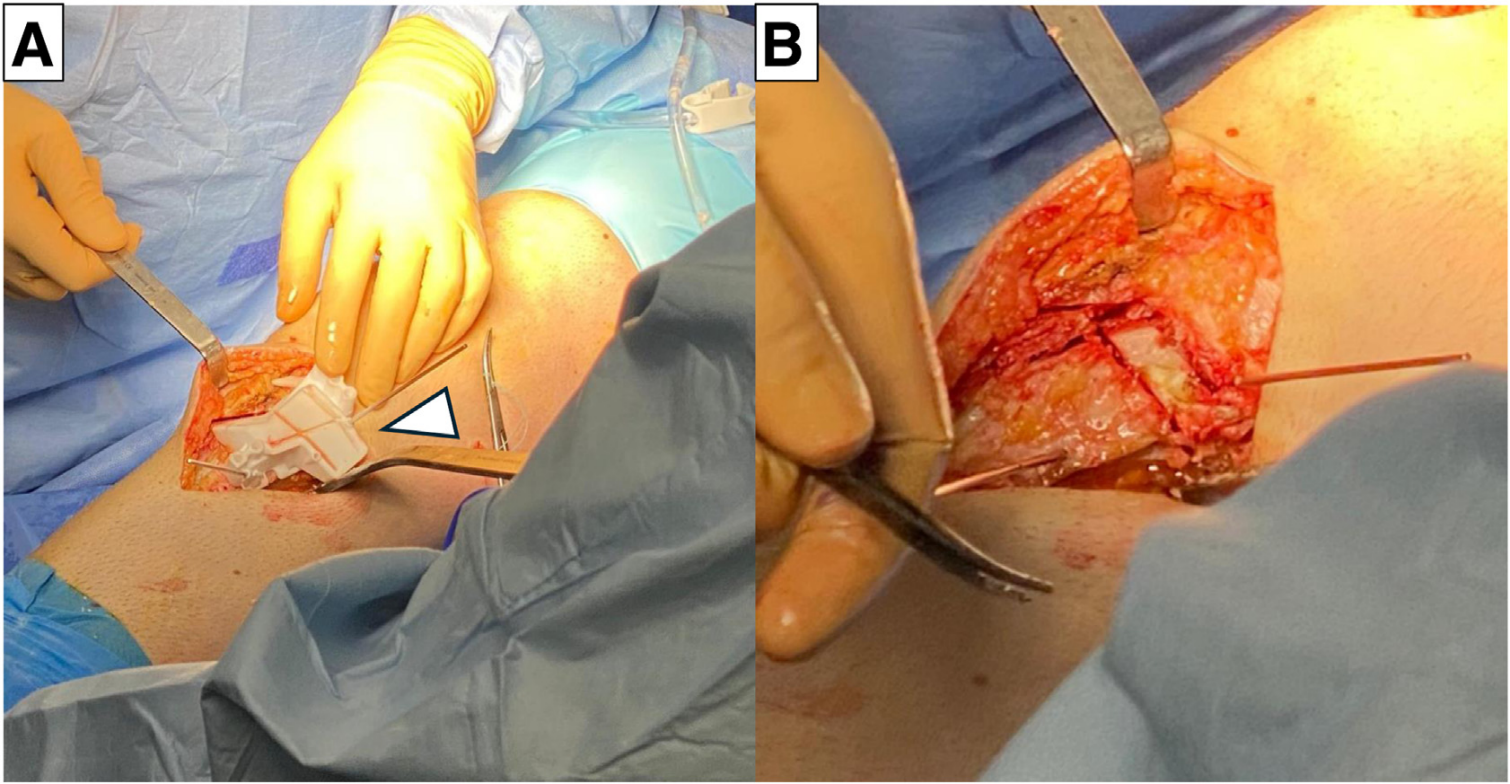
Intraoperative photographs show triplanar osteotomy is performed using the patient-specific guide.

**Fig 7:**
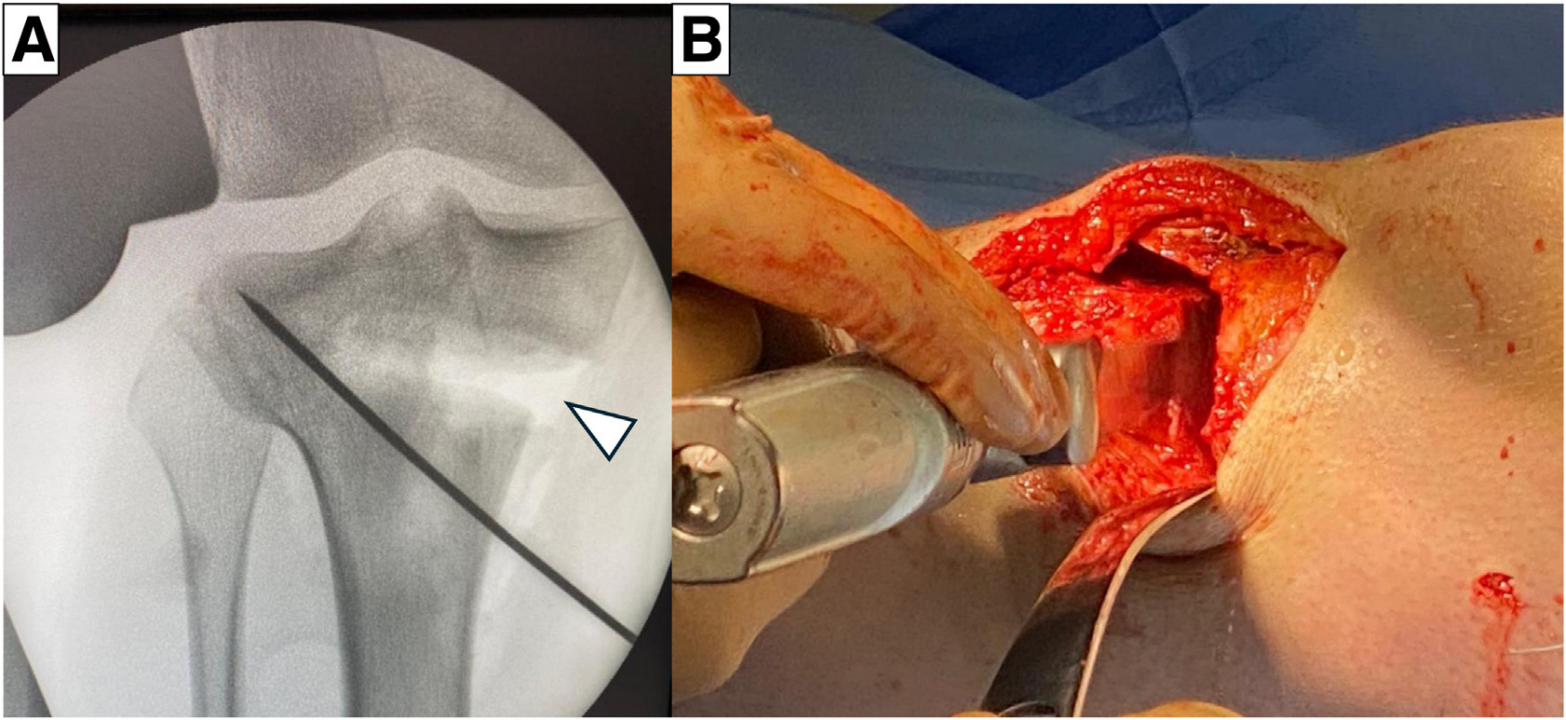
Triplane closed wedge osteotomy is performed completely and then bone wedge is removed.

**Fig 8:**
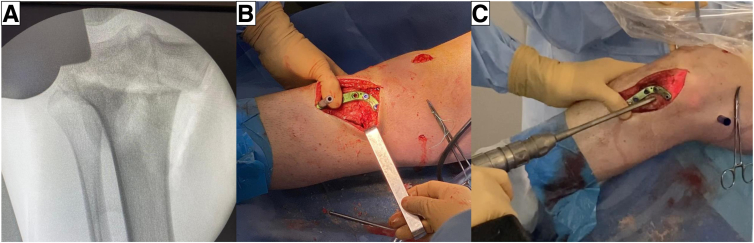
(A) Osteotomy gap is gently closed by compression to the osteotomy site. (B) Fixation is accomplished using locking plate system. (C) Tibial bone tunnel for revision anterior cruciate ligament reconstruction is created without interfering locking screws.

**Fig 9:**
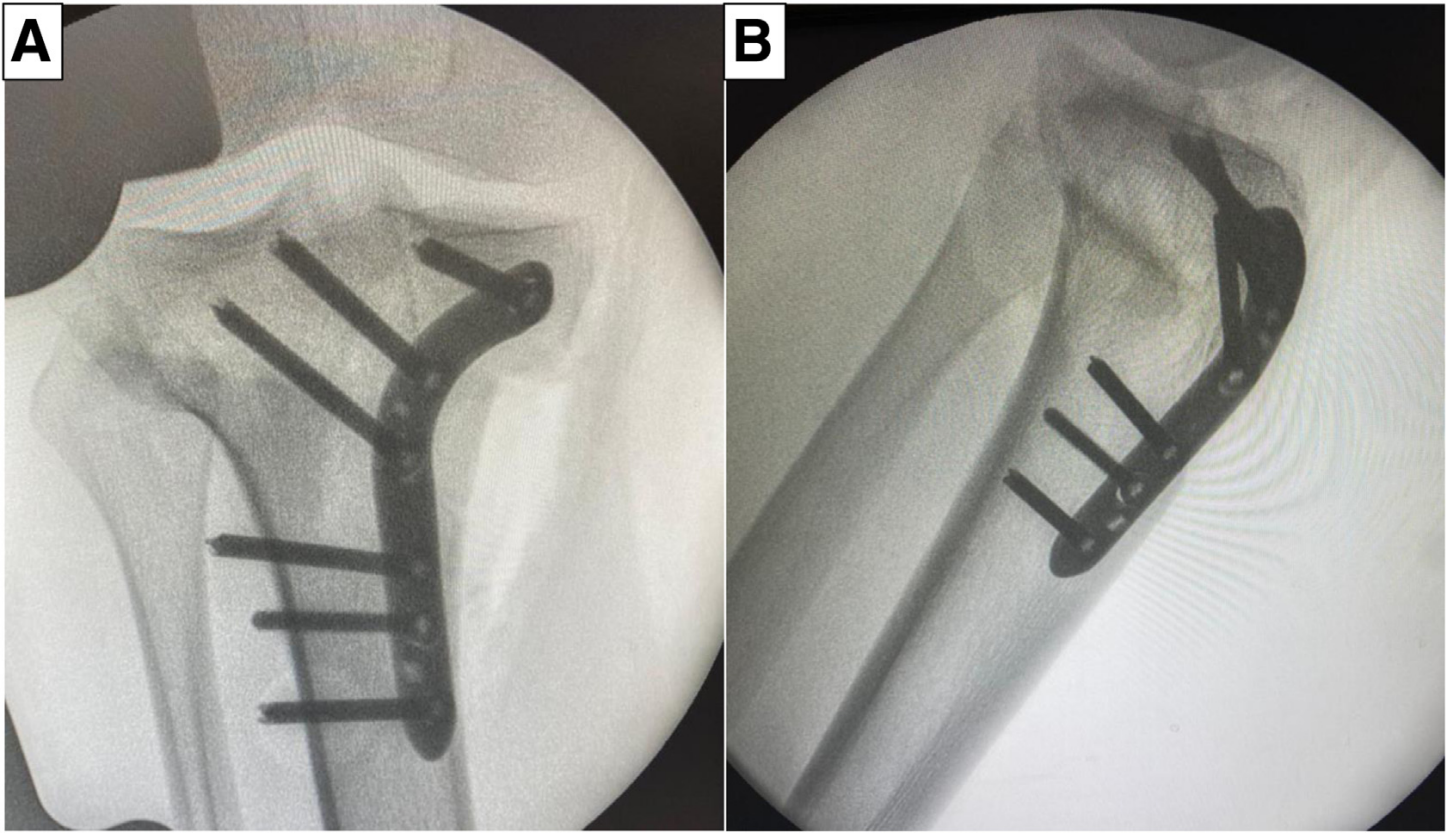
Intraoperative images under fluoroscopy shows accurate correction and fixation. (A) Anteroposterior view. (B) Lateral view.

For AMCWO, an anteromedial longitudinal incision is made over the tibial tubercle (Fig 4A). Full-thickness subcutaneous flaps are elevated. Pes anserinus is detached from the tibial insertion, and the distal superficial medial collateral ligament is released from the anterior border to the posteromedial cortex to expose the complete medial aspect of the proximal tibia. The PSI guide is placed on the medial tibial surface according to the preoperative plan and fixed with a 2.0-mm Kirshner wire (K-wire) (Fig 4B). A consistent location of the PSI guide with the preoperative plan is verified under fluoroscopy (Fig 5). Hohmann retractors are placed to protect the posterior neurovascular bundle. The saw blade is guided by the specific slots of the PSI using a combination of the reciprocating saw and osteotomes (Fig 6). The cutting PSI guide is removed to finish the osteotomy (Fig 7), and the osteotomy site is then gradually closed under manual pressure. Once intended correction is achieved, a low-profile locking plate system (Activmotion plates; Newclip Technics) is placed on the medial aspects of the tibia to secure the stability of the osteotomy site (Fig 8 A and B). Proximal locking screws are initially inserted without reducing the osteotomy site. A cortical screw is then inserted at the distal aspect of the locking plate while reducing the osteotomy site. When concomitant ACLR is performed simultaneously, a tibial bone tunnel of ACLR is created, taking care to avoid interfering with locking screws with the tibial bone tunnel (Fig 8C). The intended correction and fixation are confirmed under fluoroscopy (Fig 9).

### Postoperative Rehabilitation

**Fig 10:**
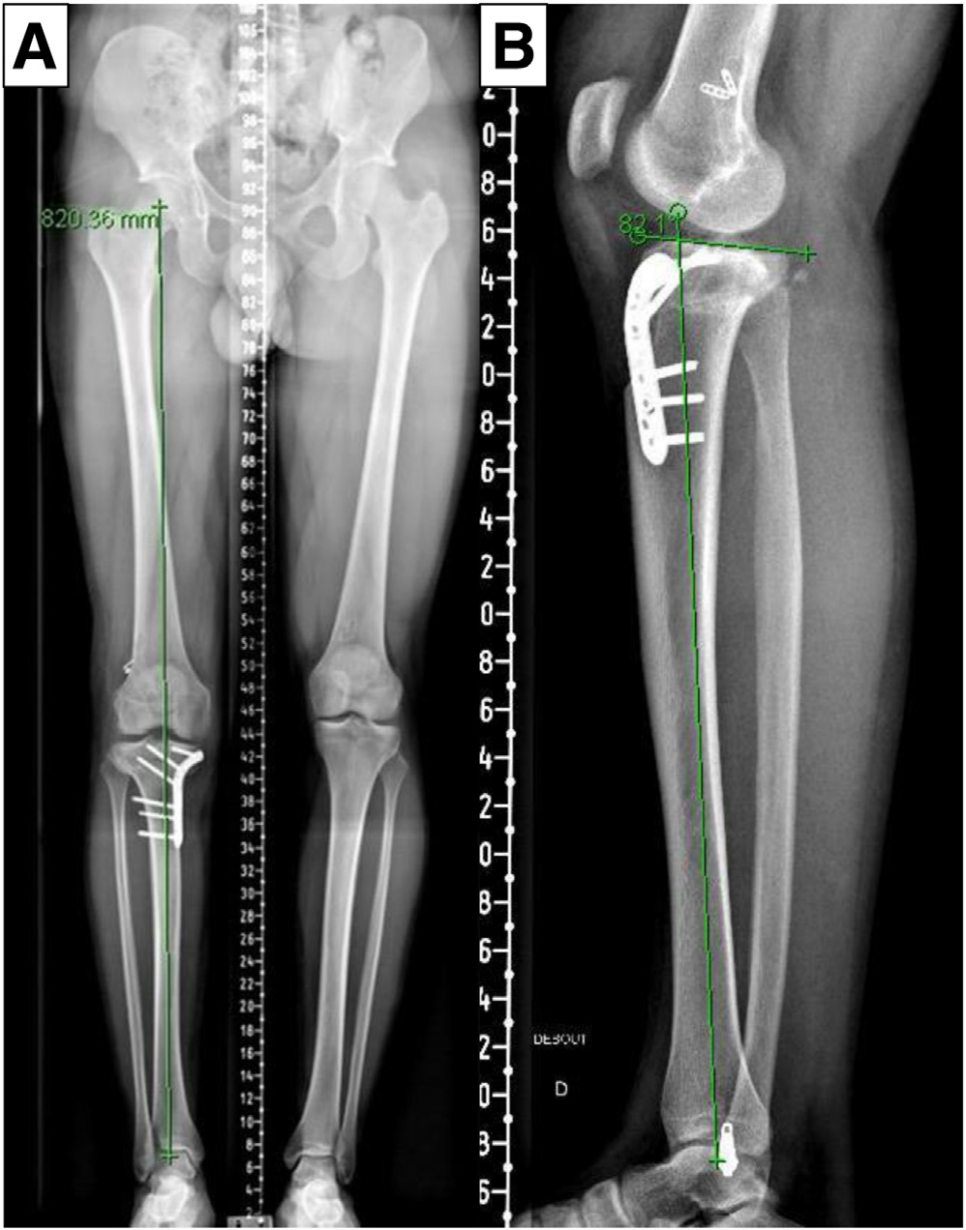
Postoperative plain radiograph. (A) Coronal long-leg radiograph shows correction to neutral alignment after the osteotomy. (B) Posterior tibial slope is reduced to 8° in the lateral radiograph.

A posterior cruciate ligament–stabilizing brace with a –10° extension restriction is recommended to prevent knee recurvatum for approximately 6 weeks. The range of motion is allowed as tolerated. Weightbearing is restricted for 3 weeks. Partial weightbearing with crutches starts at 3 weeks and progresses to full weightbearing based on radiographic healing of the osteotomy site, typically at 6 weeks. Closed kinetic chain muscle strength exercises are mainly applied for 3 months, while open kinetic chain muscle-strengthening exercises are avoided to reduce the risk of tubercle fracture until 3 months postoperatively. A postoperative radiograph is necessary to assess both coronal and sagittal alignment correction (Fig 10).

## Discussion

Excessive PTS is common in patients with multiple ACLR failures.^10^ Various ACWO techniques have been reported to reduce excessive PTS in the past decade.^3^^,^^11^ While ACWO can correct abnormal PTS, it is hard to manage coronal plane deformity. Since genu valgum usually comes from the distal femur, tibial valgum is a relatively rare condition.^12^ However, a previous review article showed that tibial valgum can occur after ACLR in pediatric patients.^8^ Combined abnormal PTS and tibial valgum could occur, especially in the setting of revision ACLR, but there is a paucity of information regarding the surgical management of this complex tibial deformity. Chan and Mohammad^13^ reported the treatment of congenital tibial valgum and abnormal PTS using a hexapod frame system. Although the hexapod frame system has the potential to accurately correct the deformity, this procedure had the risk of specific complications such as pin site infection due to the long duration of fixation wearing.^14^ Mhaskar and Saggar^15^ also reported trapezoidal wedge osteotomy combined with tibial tuberosity osteotomy (TTO) to correct excessive PTS and tibial valgum. However, TTO was associated with a high risk for either nonunion or tuberosity fracture.^16^ A triplanar proximal tibial AMCWO can correct both tibial valgum and excessive PTS without TTO, even though it is a technically demanding procedure. Although it is hard to achieve precise correction of complex deformity with the freehand technique, PSI has recently been introduced for osteotomies around the knee to improve its surgical accuracy.^17^ The use of PSI can make this complex osteotomy easier and safer. Once the optimal position of the PSI guide is verified under fluoroscopy with reference to the preoperative plan, accurate correction can be achieved even in complex deformities. In addition, AMCWO allows easy creation of the tibial bone tunnel of ACLR by preserving a sufficient amount of proximal tibial fragment and using the single low-profile locking plate system. Therefore, we believe that AMCWO should be considered a treatment option to correct the complex proximal tibial deformity of excessive PTS and tibial valgum.

This report describes the rationale, technique, and pitfalls of a triplanar AMCWO. The triplanar AMCWO technique using PSI can easily and accurately manage complex tibial deformity of combined excessive PTS and marked tibial valgum. The full list of advantages and disadvantages of this study is presented in Table 2.

**Table 2:** Advantages and Disadvantages

Advantages	Disadvantages
- The technique allows precise correction for combined abnormal PTS and tibial valgum deformity. - The complex 3-dimensional osteotomy can be performed easily and accurately by using the PSI instead of the freehand technique. - The PSI allows defining the optimal plate position, and only 1 guide is used for drilling and positioning the plate after the correction. - Preservation of the patellar tendon insertion can be achieved during the distal tuberosity osteotomy. This can help prevent unintended change in patellar height. - The low-profile locking plate allows for easy tibial bone tunnel creation in the setting of revision ACL reconstruction.	- Radiation exposure of CT scan to prepare for PSI. - Additional cost of PSI. - Risk of hinge breakage during osteotomy.

ACL, anterior cruciate ligament; CT, computed tomography; PSI, patient-specific instrumentation; PTS, posterior tibial slope.

## Disclosures

The authors declare the following financial interests/personal relationships which may be considered as potential competing interests: M.O. is a consultant or advisor for Newclip Technics, Stryker Orthopaedics, and Arthrex. K.K. is a consultant or advisor for Newclip Technics. All other authors (S.O., M.G., H.N., J.F.) declare that they have no known competing financial interests or personal relationships that could have appeared to influence the work reported in this paper.

## Supplementary Data

Video 1 Triplanar anteromedial closing wedge osteotomy of the right knee in supine position. Preoperative planning, including the standard and long-leg radiographs and computed tomography scan, is mandatory to assess the preoperative coronal and sagittal slopes, intended correction, and optimal osteotomy site. Magnetic resonance imaging is useful to assess the intra-articular pathology. Distal tuberosity osteotomy would be better to prevent postoperative change in patellar height if a patient has patella alta preoperatively. An examination under anesthesia is performed to assess the ligamentous instability. The patient is positioned in a supine position with a nonsterile tourniquet. An anteromedial longitudinal incision is made over the tibial tubercle. The pes anserinus is detached from the tibial insertion, and the distal superficial medial collateral ligament is released from the anterior border to the posteromedial cortex to expose the medial aspect of the proximal tibia. The patient-specific instrumentation (PSI) guide is placed according to the preoperative plan and fixed with 2.0-mm Kirshner wires. After optimal location of the PSI guide is verified under fluoroscopy, the saw blade is guided by the specific slots of the PSI using a combination of the reciprocating saw and osteotomes. The cutting guide is removed to finish the osteotomy, and the osteotomy site is then closed under manual pressure. Once intended alignment is achieved, a low-profile locking plate system is used to secure the stability of the osteotomy site. In revision anterior cruciate ligament reconstruction, the low-profile locking plate allows for easy tibial bone tunnel creation without interfering screws with the anterior cruciate ligament graft.
